# P-2215. Intestinal microbiome and metabolome fluctuations in patients receiving intensive chemotherapy for AML: Results from a pilot, prospective observational study

**DOI:** 10.1093/ofid/ofae631.2369

**Published:** 2025-01-29

**Authors:** Sabrina Imam, Samuel Yates, Dinanath Sulakhe, Huaiying Lin, Ashley Sidebottom, Anitha Sundararajan, Angelica Moran, Emerald Adler, Gregory Roloff, Syed Shah, Hamed Rahmani, Caner Saygin, Wendy Stock, Christopher Lehmann, Eric Pamer, Mariam Nawas, Olatoyosi Odenike

**Affiliations:** University of Chicago, Chicago, Illinois; University of Chicago, Chicago, Illinois; University of Chicago, Chicago, Illinois; University of Chicago, Chicago, Illinois; University of Chicago, Chicago, Illinois; University of Chicago, Chicago, Illinois; University of Chicago, Chicago, Illinois; University of Chicago, Chicago, Illinois; University of Chicago, Chicago, Illinois; University of Chicago, Chicago, Illinois; University of Chicago, Chicago, Illinois; University of Chicago, Chicago, Illinois; University of Chicago, Chicago, Illinois; University of Chicago, Chicago, Illinois; University of Chicago, Chicago, Illinois; University of Chicago, Chicago, Illinois; University of Chicago, Chicago, Illinois

## Abstract

**Background:**

Loss of a diverse intestinal microbiome, particularly through the depletion of anaerobic bacteria, is associated with poor outcomes in people undergoing treatment for leukemia. We present preliminary results from a pilot, prospective observational study characterizing longitudinal changes in the fecal microbiome and metabolome in patients undergoing intensive chemotherapy for newly diagnosed acute myeloid leukemia (AML).

**Methods:**

We recruited 10 patients with newly diagnosed AML who were hospitalized for intensive chemotherapy. The subjects underwent daily serum and stool collections during the admission for induction chemotherapy and periodic serum and stool collections during subsequent admissions. Metabolome profiling was conducted by targeted GC- and LC-mass spectrometry of serum and stool specimens, and fecal microbiome composition was determined by Shotgun metagenomic sequencing. Clinical characteristics, including responses to chemotherapy and development of infections, were monitored.

**Results:**

Metagenomic sequencing demonstrated marked variations in microbiome compositions between patients in our cohort. However, microbiome compositions and diversities remained stable within patients who maintained a higher prevalence of obligate anaerobes belonging to the *Lachnospiraceae* and *Bacteroidaceae* families. Moreover, despite stable microbiome compositions, we detected large fluctuations in fecal metabolite concentrations, particularly among secondary bile acids and conjugated and unconjugated bile acids, over the course of induction chemotherapy. Our results suggest that preservation of intestinal anaerobes enhances the stability of the microbiome over time, and the metabolic output of an individual’s microbiome is substantially impacted during cancer treatment.

**Conclusion:**

Studying dynamics of the intestinal microbiome and metabolome during cancer therapy will help identify signals associated with microbiome diversity or disruption. We also plan to use broad targeted and untargeted platforms to identify metabolites in stool or serum that can be used as biomarkers to correlate with microbiome compositions and to identify patients who are at risk for adverse clinical outcomes associated with disrupted microbiomes.

**Disclosures:**

All Authors: No reported disclosures

